# Neurofibromatosis Type 2: A Rare Case of Multiple Intracranial Schwannomas, Meningiomas, and Ependymomas (MISME) Syndrome and Literature Review

**DOI:** 10.7759/cureus.88131

**Published:** 2025-07-16

**Authors:** Lagtarna Hamza, Naji Yahya, Hrouch Wafa, Chouaf Loubna, Laadami Sara, Adali Nawal

**Affiliations:** 1 Neurology Department, Agadir University Hospital, Agadir, MAR; 2 NICE (Neurosciences Innovation Cognition Ethique) Research Team, REGNE (Rein Endocrinologie Gastroentérologie Neurosciences Ethique) Research Laboratory, Ibn Zohr University, Agadir, MAR

**Keywords:** astrocytoma, meningiomas, misme syndrome, neurofibromatosis type 2, nf2 gene, vestibular schwannomas

## Abstract

Neurofibromatosis type 2 (NF2) is a rare genetic disorder affecting the nervous system, primarily characterized by benign tumor formation, including bilateral vestibular schwannomas, meningiomas, and ependymomas. These tumors are collectively known as MISME (multiple intracranial schwannomas, meningiomas, and ependymomas) syndrome. We report a case of a 35-year-old woman with no notable medical or family history, who presented with progressive hearing loss and right upper limb weakness. Magnetic resonance imaging revealed multiple central nervous system tumors, confirming the diagnosis of NF2. The patient was referred to the neurosurgery department for further management; however, no follow-up information was received, and it was suspected that the patient may have sought care elsewhere. This case underscores the diverse clinical manifestations of NF2 and emphasizes the critical role of neuroimaging in the early diagnosis of the condition. A multidisciplinary approach is essential for optimal care, and future advances in targeted therapy may significantly improve patient outcomes. Clinicians should remain vigilant for the diverse presentations of NF2, as early detection and comprehensive management can positively influence the disease progression and quality of life.

## Introduction

Neurofibromatosis type 2 (NF2) is an autosomal dominant disorder characterized by the development of multiple benign tumors in the central and peripheral nervous systems, most notably, bilateral vestibular schwannomas. This condition arises from mutations in the NF2 gene on chromosome 22, leading to the loss of function of the tumor suppressor protein merlin [1,2]. Collectively, these tumors, schwannomas, meningiomas, and ependymomas, are known as MISME (multiple intracranial schwannomas, meningiomas, and ependymomas) syndrome and affect both the peripheral and central nervous systems [3].

NF2 is a rare disease with an estimated incidence of one in 25,000-40,000 individuals [4]. Approximately 30-50% of NF2 cases are known to arise sporadically due to de novo mutations, rather than through inheritance from an affected parent [4]. The disease typically presents in young adults but can manifest at any age [5]. Owing to its phenotypic variability, NF2 requires a comprehensive multidisciplinary diagnostic and therapeutic approach. The typical clinical presentation of NF2 often includes hearing impairment, balance issues, skin lesions, and visual symptoms, which can vary in severity depending on the patient. These manifestations are essential in the early identification of the disease.

In this report, we focus on the clinical presentation of a 35-year-old woman who presented with bilateral hearing loss and right upper limb weakness. The diagnosis was confirmed through magnetic resonance imaging (MRI), which revealed the characteristic tumors associated with NF2. Notably, the patient has no significant family history of NF2, which suggests a de novo mutation, a common occurrence in NF2. The absence of a genetic test due to resource constraints limits the ability to provide a definitive genetic diagnosis. This limitation has important implications for long-term monitoring, targeted therapies, and family counseling. Given the rare and varied presentation of NF2 in this patient, this case emphasizes the importance of early neuroimaging for diagnosis and the need for a multidisciplinary approach to management. Early detection is crucial for optimal outcomes, particularly in cases without a family history, where the condition may be overlooked. Genetic confirmation, if feasible, could influence long-term monitoring and therapeutic strategies, including personalized treatment options for specific NF2 mutations.

## Case presentation

A 35-year-old woman with no significant personal or family medical history presented with progressive bilateral hearing loss and right upper limb weakness. The hearing loss had been gradually worsening over the past six months, with the patient experiencing difficulty understanding speech, especially in noisy environments. The right upper limb weakness developed over the past two months, starting with mild weakness and progressively worsening, leading to difficulty with tasks such as writing and lifting objects.

On admission, she was afebrile, and her vital signs were stable. Neurological examination revealed normal mental status and speech, muscle weakness in the right upper limb (Medical Research Council (MRC) scale: 4/5 proximally and 3/5 distally in the right upper limb), and abolished bicipital and tricipital reflexes. The patient had intact light touch, pain, and temperature sensations on the upper limbs bilaterally. However, proprioception and vibration sense were mildly reduced in the right upper limb and both lower limbs, suggesting possible involvement of the posterior column function.

**Figure 1:**
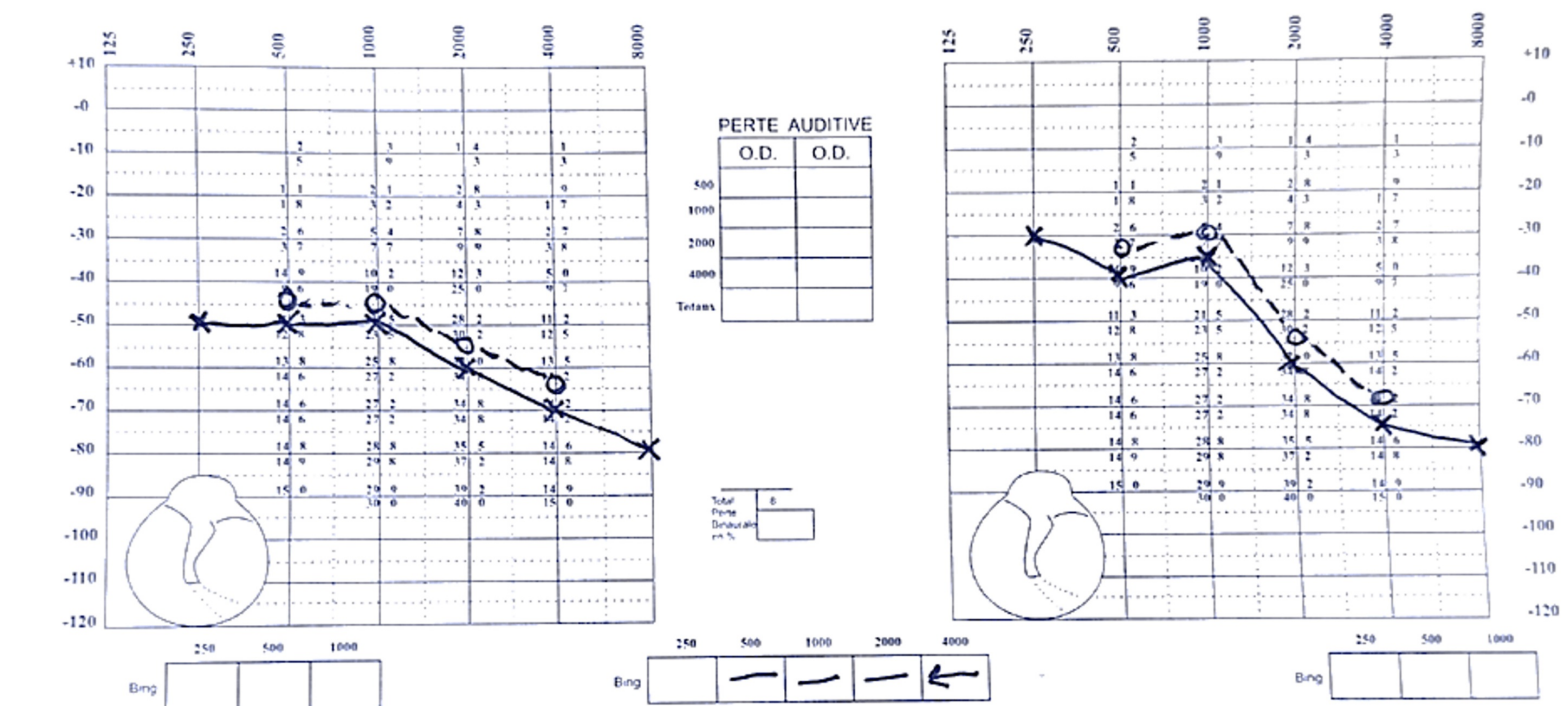
Audiometric diagnosis of bilateral sensorineural hearing loss.

Dermatological and ophthalmological examinations were unremarkable, with no cutaneous schwannomas, juvenile cataracts, or other findings indicative of NF2. Audiometry confirmed bilateral sensorineural hearing loss, consistent with the vestibular schwannomas seen on MRI. There was no significant fluctuation in hearing, but the loss was progressive over time (Figure 1).

**Figure 2:**
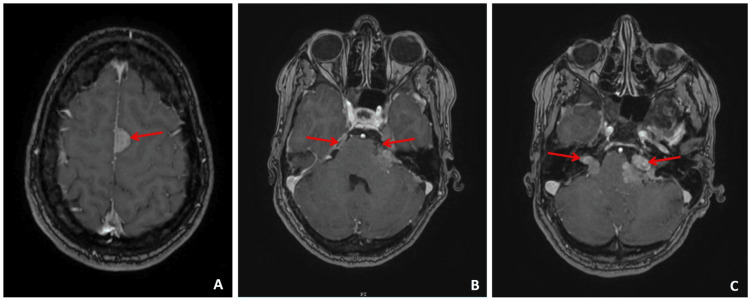
(A) Cerebral MRI axial section T1 sequence injected showing intracranial meningiomas. (B) Cerebral MRI axial section fluid-attenuated inversion recovery (FLAIR) sequence showing bilateral trigeminal neurinoma. (C) Cerebral MRI axial section T1 sequence injected showing bilateral vestibular schwannomas extending into the cerebellopontine cisterns, creating the characteristic "ice cream cone" appearance.

**Figure 3:**
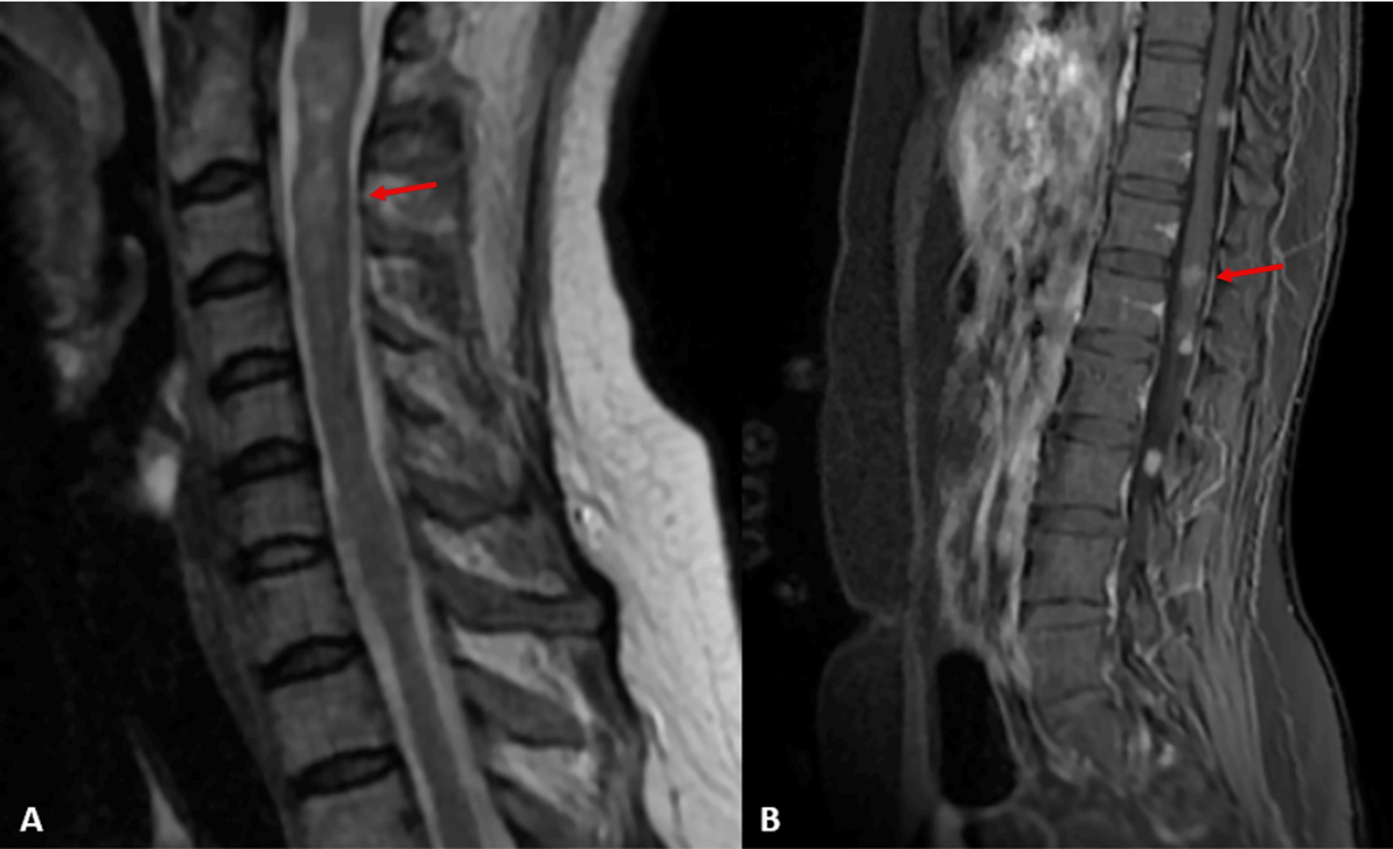
(A) MRI of the spinal cord sagittal section, T2 sequence, objectifying cervicodorsal spinal cord astrocytoma and meningiomas. (B) MRI of the spinal cord sagittal section, T1 sequence injected, demonstrating dorsolumbar spinal cord astrocytoma and meningiomas.

MRI of the neuroaxis showed bilateral vestibular schwannomas extending into the cerebellopontine cisterns ("ice cream cone" appearance), bilateral trigeminal schwannomas, a cervicodorsal spinal cord astrocytoma, and multiple meningiomas (Figures 2, 3).

While NF2 is the most likely diagnosis, other conditions, such as sporadic vestibular schwannomas and schwannomatosis, should be considered. However, the presence of multiple tumors, including bilateral vestibular schwannomas, trigeminal schwannomas, and meningiomas, strongly supports NF2. The absence of genetic testing in this case is due to resource limitations, but the clinical presentation and characteristic MRI findings are consistent with NF2, making it the primary diagnosis according to the revised Manchester criteria (Table 1).

**Table 1:** A summary table of the patient's main clinical findings, imaging results, and management plan.

Clinical finding	Details
Patient's age/sex	35-year-old female
Symptoms	Progressive bilateral hearing loss, right upper limb weakness
Neurological findings	Medical Research Council (MRC) scale: 4/5 proximal and 3/5 distal weakness in the right upper limb
Sensory findings	Mild reduction in proprioception and vibration sense in the right upper limb and both lower limbs
Audiometry	Bilateral sensorineural hearing loss
Imaging results	At the brain level: Presence of multiple brain lesions with varying locations and sizes
	Bilateral vestibular schwannomas measuring 13 x 9 mm on the right and 19 x 17 mm on the left
	Trigeminal neurinoma measuring 10 x 5 mm on the right and 13 x 7 mm on the left, with extension into the Meckel's cave
	Left cerebellopontine angle (CPA) meningioma measuring 19 x 9 mm, located posterior to the left acoustic neurinoma
	Meningioma of the cerebellar tentorium measuring 6 x 4 mm on the right, anterior to the transverse sinus, and 10 x 7 mm on the left
	Right temporal meningioma measuring 10 x 6 mm, left frontal meningioma measuring 8 x 4 mm, and left parasagittal meningioma measuring 15 x 10 mm
	Multiple linear and pseudo-nodular meningeal contrast enhancements in the right upper parasagittal and left frontal regions
	At the spinal level:
	Multiple intramedullary lesions (astrocytomas) with the following locations and sizes: C2: 13 x 10 mm; C3: 15 x 7 mm; C6: 6 mm; C7: 5 mm; D3: 4 mm; D7: 3 mm; D8: 3 mm
	Anterior meningioma at D10 measuring 9 x 8 mm and posterior meningioma at D11 measuring 7 mm
	Multiple neurofibromas of the conus medullaris and cauda equina nerve roots measuring between 6 mm and 11 mm
Management plan	Referral to the neurosurgery department for further management, symptomatic monitoring, and tumor management

The patient was referred to the neurosurgery department for further management following the diagnosis of NF2. However, due to the absence of follow-up information and the possibility that the patient sought care at another facility, the outcome remains unclear. Additionally, the limited resources and infrastructure of our hospital impacted our ability to provide a comprehensive diagnosis and personalized treatment plan. Despite these constraints, a multidisciplinary approach involving tumor monitoring and symptomatic management is essential for optimizing treatment outcomes.

## Discussion

NF2 is 10 times less common than NF1, with an estimated incidence of one in 25,000-40,000 individuals, and the term MISME syndrome is synonymous with NF2 [1,2]. Although NF2 typically presents in young adults between the ages of 18 and 24 years, it can manifest at any age, and diagnosis is usually confirmed through a detailed clinical examination and imaging, particularly MRI. In this case, a 35-year-old woman presented with bilateral hearing loss and right upper limb weakness, which were attributed to vestibular schwannomas and intramedullary tumors. This case highlights the atypical presentation of NF2 in a patient without a family history, suggesting a de novo mutation, which is often more difficult to diagnose, particularly in resource-limited settings where genetic testing may not be available.

NF2's variable symptoms complicate the diagnosis. Hearing loss, as seen in our patient, is common in approximately 60% of cases; other manifestations, such as visual impairment, facial paralysis, and spinal involvement, can also occur. The presence of vestibular schwannomas causing progressive deafness and balance issues is a characteristic feature of NF2 [1,2]. However, in settings where genetic testing is not possible, clinical presentation and MRI are critical for diagnosing the condition. In our case, the absence of genetic confirmation due to resource constraints presented a challenge for prognosis, risk assessment, and personalized treatment planning.

MRI, especially with gadolinium contrast, plays a pivotal role in diagnosing NF2, as it allows for the detection of tumors, their characterization, and aids in near-definitive diagnoses in most cases [2,6]. Vestibular schwannomas exhibit distinct features on magnetic resonance imaging (MRI) and are located in the internal auditory canal, where they are often dilated [7]. Large tumors can also be observed at the cerebellopontine angle, forming a typical ice-cream cone appearance. These lesions are hyposignal T1 and hypersignal T2, with high-contrast uptake [6,7]. Larger tumors may adopt cystic forms. There is no correlation between tumor size and the degree of hearing impairment [7]. Meningiomas are the most frequent type of extra-axial tumor found in NF2 patients. Therefore, NF2 should be considered in the differential diagnosis of meningiomas in pediatric patients [8,9]. On MRI, meningiomas commonly appear as dural-based masses, exhibiting isointense signals on T1-weighted images and iso- to hyperintense signals on T2-weighted images, along with strong contrast enhancement and a distinctive dural tail sign [3,8,9].

NF2 patients can also present with multiple spinal tumors, including schwannomas, meningiomas, and ependymomas, all of which can be diagnosed using MRI. Schwannomas are the most frequent type, usually originating from the dorsal roots. They often exhibit a characteristic dumbbell shape associated with widening of the neural foramen and marked contrast enhancement [7]. Meningiomas present as extramedullary lesions, most commonly located in the cervical or thoracic spine, and typically show contrast enhancement [3]. In contrast, ependymomas are intramedullary tumors that cause spinal cord enlargement and are often accompanied by hemorrhage, cystic changes, and variable contrast enhancement [3]. Astrocytomas are rare, with most being low-grade tumors [7].

The management of NF2 patients requires a multidisciplinary approach involving oncologists, neurologists, neuroradiologists, ophthalmologists, geneticists, and neurosurgeons [3]. If the initial scan reveals no brain tumors, a follow-up MRI can be conducted every two years. However, if a tumor is identified, MRI should be performed twice within the first year, followed by annual surveillance scans [3,8]. Surgical intervention is recommended for patients with symptomatic vestibular schwannomas. Meningiomas are also treated with surgery, while radiation therapy is an option for patients who are not suitable surgical candidates. Spinal cord ependymomas, which are typically low-grade tumors, are generally monitored clinically, with surgery reserved for symptomatic patients [1,3]. Several studies have documented that tumor shrinkage, particularly in vestibular schwannomas and meningiomas, has been observed in patients receiving bevacizumab, with some tumors reducing in size by approximately 10-20% after several months of treatment. However, serious side effects have been reported; therefore, the judicious use of bevacizumab for symptomatic management is recommended [10].

This case adds new knowledge by highlighting the importance of radiological criteria for diagnosing NF2 in the absence of genetic testing. The patient’s atypical presentation, being 35 years old and without a family history, also emphasizes the need for healthcare providers to consider NF2 in patients with unexplained hearing loss and neurological deficits, even without familial predisposition. Furthermore, this case illustrates the therapeutic challenges in resource-limited settings, where genetic testing and advanced personalized treatments may not be accessible to patients. In such settings, early neuroimaging, a multidisciplinary approach, and judicious use of available therapies remain the cornerstones of effective management.

## Conclusions

NF2 is a complex disorder that requires early diagnosis and coordinated care. This case illustrates the varied manifestations of NF2, including bilateral vestibular schwannomas and CNS tumors, with the patient presenting with hearing loss and right upper-limb weakness. MRI confirmed the diagnosis, but genetic testing was not performed because of resource limitations, highlighting the challenge of diagnosing NF2 without genetic confirmation.

In settings with limited resources, MRI should be considered the primary diagnostic tool when NF2 is suspected, especially in patients with unexplained hearing loss or neurological deficits, even in the absence of a family history. Surgical management and therapies, such as bevacizumab, offer potential benefits for symptomatic patients. A multidisciplinary approach is essential for optimizing care and improving patient outcomes. This case underscores the need for early intervention. When genetic testing is unavailable, healthcare providers should rely on clinical presentation and imaging to guide diagnosis and management.
